# ‘It’s communication between people who are going through the same thing’: experiences of informal interactions in hospital cancer treatment settings

**DOI:** 10.1007/s00520-023-07900-6

**Published:** 2023-07-03

**Authors:** Matthew P. Grant, Jennifer A. M. Philip, Luc Deliens, Paul A. Komesaroff

**Affiliations:** 1 Palliative Nexus Research Group, Department of Medicine, University of Melbourne, Melbourne, Australia; 2 Department of Palliative Medicine, St Vincent’s Hospital Melbourne, Melbourne, Australia; 3 Centre of Expertise in Palliative Care Utrecht, Department of General Practice, Julius Centre, University Medical Centre Utrecht, Utrecht, The Netherlands; 4 End-of-Life Care Research Group, Vrije Universiteit Brussel (VUB) and Ghent University, Brussels, Belgium; 5 Monash University, Melbourne, Australia

**Keywords:** Communication, Informal interactions, Cancer treatment, Palliative care

## Abstract

**Purpose:**

In hospital settings, patients, visitors, and staff engage in many interactions outside formal clinical encounters. Whilst many of these may be inconsequential, others contribute significantly to how patients and their carers experience cancer and its treatment. This article aims to explore the experiences and significance of interactions that occur outside formal clinical encounters in hospital cancer treatment settings.

**Methods:**

Semi-structured interviews were conducted with cancer patients, carers, and staff recruited from two hospital sites and cancer support groups. Hermeneutic phenomenology informed lines of questioning and data analysis.

**Results:**

Thirty-one people participated in the study: 18 cancer patients, four carers, and nine staff members. The experiences of informal interactions were grouped into three themes: connecting, making sense, and enacting care. The participants described how these encounters allowed connection with others in the hospital spaces, facilitating a sense of belonging, normality, and self-worth. Through these interactions, individuals participated in making sense of their experiences, to better anticipate the decisions and challenges that might lie ahead. By connecting with other individuals, they cared for others and felt cared for themselves, and were able to learn from, teach, and support each other.

**Conclusions:**

Outside the confines of the clinical discourses participants negotiate terms of engagement, sharing of information, expertise, and their own personal stories that they may employ to contribute to the individuals around them. These interactions occur within a loose and evolving framework of social interactions, an ‘informal community’, in which cancer patients, carers, and staff members play active and meaningful roles.

**Supplementary Information:**

The online version contains supplementary material available at 10.1007/s00520-023-07900-6.

## Introduction

In hospital settings, patients, visitors, and staff engage in many interactions outside formal clinical encounters. In his work on palliative care, Allan Kellehear describes how hospitalised patients spend approximately five percent of their time in the presence of health care professionals at the end of life [1]. Social research has largely focused on this brief period that patients are with health care professionals, and even then, only that portion of it that occurs within the boundaries of the clinical discourse, paying little attention given to other elements of hospital care that may not be recognised as being part of the formal care process. However, these informal interactions are a universal feature of the hospital space and the formal care process [2]. They may take the form of a seemingly inconsequential greeting in the elevator, a discussion about weather in the corridor, or a jocular chat about football whilst preparing for chemotherapy [3]. Equally, they may involve profound reflections: children discussing their dying parents on the hospital ward, patients sharing their experiences of treatments, and health professionals, patients, and families sharing their personal stories and discussing issues and concepts that may be challenging to address within the confines of the medical discourse [4, 5].

These informal interactions occur as an inherent feature of hospital care, within and adjacent to formal health care processes [6]. In the setting of cancer treatment, patients, carers, and staff members are engaged in longitudinal care, with regular chemotherapy and radiotherapy, clinical appointments, and hospital admissions through which formal treatment relationships — some of which become long-lasting — are developed. However, it remains unclear how the informal elements are experienced and negotiated by patients within such treatment relationships. Additionally, as a result of their regular and intense treatment schedules, patients and carers come frequently and repeatedly into contact in settings of common experiences of cancer, which may both facilitate interactions and support the development of ongoing relationships [2, 7].

Whilst there is a considerable body of literature examining the role of cancer support groups, relatively few studies have examined interactions between patients in the hospital setting, and the ways in which they may contribute to social supports, expand knowledge of illness and its treatment, and enhance care [3, 6–10]. Whilst it is recognised that patients often support each other through changes to their health and social circumstances [11], how family and staff experience these interactions remains incompletely understood. Anecdotal evidence suggests that staff members may be an integral part of these relationships, and the apparently trivial social exchanges that occur during chemotherapy treatment may contribute significantly to patient well-being, support and care, and may even enhance the formal work of health professionals.

In view of the lack of an established, systematic framework for describing the informal domain of health care, it may be helpful to clarify some of the concepts used in the present analysis. The term ‘interaction’ is used to describe a temporally circumscribed event of social engagement involving two or more persons [12]. ‘Clinical discourse’ is used to refer to the rigorous, formalised, systematic modes of enquiry, observation, and documentation of interactions with patients applied by health professionals in the setting of hospital care [5, 13]. This clinical discourse incorporates certain key elements that define its time course, order, and structure, including the physical examination, the hierarchy of knowledge, and recommendations of the practitioner, which together encompass the ‘formal’ clinical encounter [14, 15]. The term ‘informal interaction’ is used to describe those interactions that occur outside the formal or deliberate clinical encounter. Informal interactions may occur within the clinical consultation but if they do so they remain outside the formalised dialogues and agenda of clinical investigation and management [13].

The aim of this study is to understand the experiences and significance of informal interactions in cancer treatment settings for the various participants, including patients, family carers, and health care staff.

## Methods

This qualitative study utilised semi-structured interviews to explore the perceptions of informal communities in cancer treatment settings, informed by phenomenological methods [16]. Reporting was conducted according to the Consolidated criteria for reporting qualitative research (COREQ) checklist [17]. Ethics approval was obtained through the St. Vincent’s Hospital Melbourne Human Research Ethics Committee (#LNR/17/SVHM/59).

### Design and procedure

Study participants were patients, family carers, and staff members, with data collected between May 2017 and September 2018. Eligibility criteria included age over 18 years and ability to speak English and provide consent.

Participants were recruited from the cancer treatment settings of a tertiary hospital network (chemotherapy day unit, oncology, haematology, and two palliative care wards) and cancer support groups. Patients and carers were initially sampled via a convenience sampling approach, with subsequent purposive sampling based on the cancer treatment approach adopted (curative, chronic, or palliative). Purposive sampling of staff ensured representation across roles (doctors, nurses, allied health, administrative) and sites of work (chemotherapy day unit, oncology ward, palliative care). In addition, a sample of patients was recruited through cancer support networks using a convenience and then snowball sampling approach.

One of the researchers (M. G.) approached potential participants, providing an overview of the research, an invitation to participate, and a copy of the information sheet. Written consent was obtained for all patients, staff-members, and carers, with verbal consent available for support group members to facilitate telephone interviews. Interviews were conducted by M. G., a male palliative medicine physician with experience and training in qualitative research, who was not involved in the care of the participants.

### Data collection

Demographic and clinical (for cancer patients) characteristics were collected, including gender, age (patients), cancer type (patients), treatment intent (patients), and clinical role (staff). All interviews were semi-structured in nature and were adaptive to the conversation and experiences of the participants, following an interview guide with lines of questioning (see supplementary files, Table 1). The interviews were audiotaped and transcribed verbatim and were supplemented by the researcher’s field-notes.

The interview focused on participants’ most recent experiences of interactions within the cancer treatment setting. Participants were asked to recount these experiences and explore their significance. Questioning followed eight lines of enquiry, developed from review of the literature examining patient-patient interactions [3, 6, 7, 11, 18, 19]. The data relating to each line of enquiry were reviewed throughout the study and data collection ceased when all eight lines of enquiry were saturated [20].

### Data analysis

Data were analysed by the entire research team, conducted as an iterative and multiphase process alongside data collection [21]. The approach was informed by the principles of hermeneutic phenomenology, examining the experiences shared by the participants as communicating meaning that was reflective of social and environmental context and could be interpreted on multiple levels [16].

The first step in the interpretative process involved familiarisation with the data through listening to and transcribing the interviews, reading, and re-reading these transcriptions [16]. All interviews were analysed together, as from an epistemological viewpoint, the relevance of these formal role categories in the informal domain was uncertain. From this familiarisation with the data, initial codes were generated. The research team then attempted to construct thematic categories that reflected the content of the data. The research discussions and coding arrangements were documented and reflected upon throughout the analytic processes. These processes were cyclical, with the research team discussing their assumptions, pre-conceptions, and their influence on the interpretation of the data [16]. These processes of interpretation and categorisation of the data were repeated until a structure was formed that faithfully represented the data. NVivo software (QSR International, version 12) was employed to assist with cataloguing data.

## Results

Thirty-one participants were interviewed for this study (see supplementary file, table 2), including 18 people with cancer, of whom five were recruited through cancer support groups, four family carers, and nine staff members. Eighteen of the participants were female. Interviews ranged in length from 18 to 93 min, with a mean time of 38 min.

The experiences of these interactions for the individuals involved were understood through three themes: “connecting”, “making sense”, and “enacting care”. These themes were described by all participant groups. Table 1 illustrates the personal experience of these interactions.

**Table 1:** Example of data describing experience of informal interactions

*Oh, it’s just general. “How are you? How are you feeling? How’s your sickness going? Have they worked out the right meds for you now? And just how’s your day? Have you seen the kids? Have you been out to a big restaurant?” I mean, it’s just all … a bit of medical and general stuff in between. Mostly, how they feel and what they’re up to, where they’re going and what they’ve got planned for the weekend. It’s all just like a normal group, but they’re all hospital patients* Shirley^*^ seems to get a lot … she gets a lot of knowledge, because she’s got … Oh, shit. What has she got? Yeah, whatever’s she got, plus a mental illness, so she’s not a very easy learner. She’ll ask me a lot of just general questions. Just about the weather sometimes, or … How could you put it with Shirley? *She’ll ask you anything and everything. Whatever you teach her or tell her, she writes down. Then she’ll go home and she’ll learn what you’ve taught her. Then she’ll come back and tell you what you’ve taught her. That’s how I do it with her. Pen and paper. Because she really can’t remember. Her depression and all that is very, very, high. But that’s how I work with her* *She just went one day and got pen and paper and said, “Right. Let’s go. This is what I need to know. Can you help?” I’ll help with whatever she asks. Who knows what it’s going to be. If I can answer it, I’ll answer it. If not, I’ll go find out for her, then I’ll come back and tell her. That’s how we do that* Johan^*^ just gets friendship because he lives in the country^*^. He just gets friendship, and a bit of knowledge. “With your medications and that, go easy on it. Don’t OD on that stuff. Don’t take too much.” Because he might say, “Oh, I took six of those,” and I’ll go, “Why for?” “Oh, the buzz.” “Yeah, but when the buzz runs out, you run out, don’t you?” Just stuff like that. Just your general stuff. Be careful. But he gets the friendship. A little bit of learning knowledge. That’s how he goes Dan^*^ is pretty much the same. He’s on a learning curve because he’s only a pup. Yeah, so pretty much, we just all learn off each other. We try and learn off each other, so we can help each other next time around. That’s about it *Yeah, it’s a big support group. That’s what it is. It’s just an in-house support group. Then we go home, within a week or two of everybody getting better, then we start our out-house support group. That’s when we start meeting up*
- Patient, palliative care ward

^*^Names and places have been altered

### Connecting

The participants described informal interactions as a space where they could connect to others outside the formal clinical encounter. Through connecting with other individuals, they experienced numerous meanings, including strengthening of relationships, personal worth, normality, and perceptions of commonality or distance.

#### Belonging

Participants described feeling connected to many of the other participants, using metaphors such as ‘family’, ‘support group’, and ‘network’, and referred to the relationships as ‘friendships’ or being ‘mates’. Being connected to others enabled them to feel supported and their personal situation understood.

*It’s the sort of thing that maybe in ten years time, if you met up with them you’d make a celebration out of it, because you learnt a lot about them*. (patient, chemotherapy day unit)

#### Commonality and difference

The participants, in particular the patients, described many common elements they shared with other people in the informal domain. These commonalities were frequently related to the illness, their experiences of treatment, or attending the same environment. However, these interests were frequently social and personal, including football, a similar cultural background, or children of a similar age. These shared experiences and interests were frequently at the base of the development of relationships.

*When you meet another patient, it is definitely a support group. When someone comments about my hat or something, its communication between people who are going through the same thing. It is a positive thing we go through, but because of a negative event.* (patient, chemotherapy day unit)

These interactions also provided forums for the participants to discuss or demonstrate difference, which could be positive or negative. A remarkable story of cancer treatment or life experience often stimulated interactions, enabling others to access to new information and understandings. Other participants perceived that they were different to the others in the hospital environment on a range of personal, illness, or social factors. They identified this as a reason why they did not engage with others in interactions, or only in a superficial manner.

*But a lot of them are sad sacks. I can’t be a sad sack, so I didn’t stay. Yeah, nah. Nah. I get sad days, don’t get me wrong, but I can’t be a sad sack. Nup.* (patient, palliative care unit)

#### Feeling valued

Connecting with others allowed the participants to feel valued and appreciated. Sharing personal stories and information with another person seemed to establish this perception of value, as people felt respected as a result of both being listened to and having personal information shared with them.

*It’s valuable to be just to be recognised as: oh, he’s not just my doctor, he’s another human being.* (staff, haematologist)

*Well, a sense of connection, obviously. Yes, they’re good to have, those kind of conversations. I like talking about books and movies a lot…I don’t need to say anything because they pick it up. They come in with a pile of books.* (patient, hospice)

#### Being normal

The informal interactions frequently took the focus away from cancer, instead moving attention to other aspects of the participants’ lives. In these conversations, the foci were frequently social events, food, amusing narratives, and special interests or life experiences. These interactions inserted elements of social and personal identity into the hospital space, beyond the role as a cancer patient, nurse, or pastoral carer. Participants described this as allowing them to feel normal, forget about their cancer, and bring the outside world into the hospital environment.

*All of a sudden they weren’t so reduced to their symptoms. In this dynamic, they could be themselves, how they were outside of our hospital walls.* (staff, psychologist)

*It’s a bit easier to talk to people that’ve got the same problems as you. When they said talk to somebody that’s so-called normal, of course they don’t have a clue what you’re talking about because they haven’t been through that experience.* (patient, chemotherapy day unit)

### Making sense

The interactions process facilitated participants to engage in sense making around their experiences, cancer and treatment, and their understanding of what the future may be. Through learning of patient’s and carer’s experiences, staff gained a much fuller and nuanced understanding of cancer treatment and the patient.

#### Sharing experiences

Experiences were shared of illness, stories of life, and personal history. These discussions frequently focused on the experiences of cancer, often conveyed in narrative form. Although staff did not, for the most part, have their own personal experience of cancer, they might relay the narratives they had heard from patients to other patients and carers. Carers shared their own stories, and the effects of cancer diagnosis and treatment on their life and health.

*If you find people in a relatively small area and they were getting the right treatment, and at night-time ‘cause you’re sitting there for an hour. “Oh, where are you from? How many treatments have you had?” Before you know it, “Do you mind giving me a number and we can swap notes.” Before you know it, you talked about it.* (cancer support group member)

*You know that those girls understand how shit the chemo really is ‘cause they’ve had it.* (patient, oncology ward)

#### Comparing

The participants engaged in the sharing of experiences through contrasting and comparing. This could be done as an individual, comparing others’ experiences to their personal situation. One participant described a group of patients that frequently met whilst at the hospital when they compared and sought to make sense of experiences.

*It’s always nice to know what’s happening with other people — to compare yourself and see where they are at. It gives you a bit of an idea of where you are. Sometimes you are passing on the goods, you know.* (carer, chemotherapy day unit)

#### Unpredictability

The view of the unpredictable nature of cancer was commonly discussed. The participants reported that the medical discourse around cancer was often narrow and did not consider the uncertainty that was part of the experience of diagnosis and treatment. Through sharing personal experiences, the participants explored a range of potential outcomes related to cancer and how it may affect their future. Some participants perceived the uncertainty in these discussions as being confronting, making them feel scared and more pessimistic about their future. For others, these experiences promoted greater understanding, and as a result greater control of their personal situation.

*I find a lot of times, people will go and say I’ve had that drug and they didn’t like it and that’s fine. That’s good for them to be informed because that shows to me that they actually want to be involved. But, at the same time, it’s a bit of a double edge sword.* (staff, haematologist)

One important domain described by the participants was the experience of loss and dying. This topic was perceived as distressing, but also one they explored, noting that understanding others’ experiences could inform their own approach.

*He’s in a better place. That’s it. He’s home now. He’s better. He can’t hurt anymore, so he’s happy. We’re happy.* (patient, palliative care ward)

### Enacting care

The participants described that through these interactions, they cared for others and were cared for.

#### Emotional and social support

Through these interactions the participants discussed how they supported others, emotionally and socially. This might be achieved through listening to them, providing support, or displaying empathy. These qualities of support were present within these interactions even when there were no direct references to emotionality or suffering. These acts of connecting and sharing, such as discussions about football or holidays, were perceived as being key social processes that supported their well-being.

*But if we make it a good day, like if we make it positive, they’ll go away happy.* (staff, administration)

*As I said, to meet other people with metastatic cancer and how they’re coping with it, what they do, what they kind of think…. I still find it so supportive, as I did. Good for my emotional and mental health, in having that context.* (patient, hospice)

#### Teaching and learning

Participants described using their own information and experience to provide advice to others within these interactions. They thus became an advisor or teacher, particularly for those less experienced patients and carers. This included discussing the different clinical trials, advising on the use of particular medications, or how to frame a question to the doctor in a specific way to arrive at the desired outcome. It could also include sharing advice on the best local restaurants and football tips. The process of learning and teaching was valuable to expanding their own knowledge and helping others.

*Some will ask about different medications that they’ve seen, read, or heard of, or they’ve got but they don’t fully understand ‘em, so you try and find some way to help them understand what they do and what they’re going to do for you.* (patient, palliative care ward)

*I am not entirely at the mercy of the medical people who — they do have success and they do cure people, but there are lots of consequences along the way. There is also that feeling of self-determination that I can, I don’t have to just put myself in the hands of other people — I can do something about it.* (support group member)

## Discussion

This study describes, from three different perspectives (people with cancer, their family carers, and hospital staff), a range of experiences of informal interactions in hospital cancer treatment settings. The significance of these experiences is similar to that commonly associated with many other social interactions, such as connecting with others and gaining a sense of belonging, self-worth, and normalising their experiences [22]. Through sharing and comparing their experiences, and thereby engaging with uncertainties that were likely to be ongoing, participants were able to develop a deeper appreciation of their situations and thus achieve a greater sense of agency. Participants engaged in learning and care through these interactions, which enriched their capacity to understand their experiences and treatment, and supported development of these relationships [23, 24]. These results are consistent with those of previous studies describing the experiences of cancer support groups, where support, learning, and care occur as social processes through the development of interpersonal relationships, and where the understanding of others’ experiences facilitates the ability to shape meanings of illness and to enact change [25–27].

A striking finding was the significance of the social connectivity, which was apparent through all three themes of the results. The participants used various names to describe these bonds, such as support group, ‘community’, ‘friendship’, and ‘companionship’. These processes of relating to others enabled individual participants to engage in acts of meaning creation, to enact care, and to make sense of illness and treatment. This connectivity was often facilitated by further conversations outside the clinical discourses of the formal domain in which interactions were negotiated between individuals independently of (yet likely still influenced by) rigid role and discourse structures [13]. In this informal discourse, the participants had a sense of greater agency over the content and direction of social relationships, in which they could exercise control over the information that was exchanged and seek support if needed [26]. The content of these discussions often focused on personal stories and experiences, relating information that was indivisible from the storytellers and their identities. The sharing of personal information facilitated the development of close relationships, in which participants interacted freely and equally, consistent with the existing literature about patient-patient interactions [3, 18, 28–30]. This is in marked contrast to formal care relationships, which are primarily experienced in accordance with their outcomes, focused on meeting the informational and care needs of the patient, and the professional needs of the health practitioner [31, 32]. This supports the conclusion that social connectivity is a structural element of these interactions and, through enacting care, learning, and sense-making, can strengthen the bonds of these relationships. The interactions exhibit recurring and patterned processes, which we propose is appropriately understood as constituting a novel, “informal” kind of “community” [2, 33].

The communities established through this interplay of informal interactions involving cancer patients and their carers are fluid and dispensable — or “liquid”, as described by the sociologist Zygmunt Bauman [34, 35]. Bauman’s late-modern concept of community is in contrast to those enduring and immovable forms of community, such as the family and village, that are a feature of modernist and pre-modernist sociology [34, 36]. The greater connectivity of the current age allows individuals to interact through multiple social assemblages that may be geographically dispersed and culturally diverse, and are realised either face-to-face or via a range of media [35]. This fluidity allows individuals not only to engage contemporaneously in a great many communities, but also to move seamlessly between them. The “informal communities” thereby established may be transient or enduring, and embrace both intimate relationship and ones lacking in depth [34]. Similarly structured communities have been described in occupational settings that arise spontaneously in response to stimuli such as common experiences, values or beliefs, or shared goals or organisational inadequacies [37, 38]. Snowdon and Schulte describe how these informal communities function parallel to, and in support of, formal organisations and through their fluidity are able to address needs for which the rigidity of formal structures constrain their response [37, 38]. Our results, which emphasise the centrality of communication processes for the establishment of meaning, providing support, and realisation of objectives [37, 39], strongly support these conclusions.

### Implications for practice, policy, and research

Our study suggests that the informal interactions in which patients and carers engage in the cancer treatment settings we have described are experienced as patterned, stable social processes with clearly identifiable dynamics and a potent meaning-creating purpose. These informal communities constitute an important — although hitherto largely invisible — component of the therapeutic context; the exact roles of which, and the extent to which they can be shaped and directed, remain to be fully elaborated.

This research focuses on the retrospective experience and meaning of informal interactions, and further research would seek to characterise in more precise detail the ways in which the communities are formed, develop, and disperse over time, and how they interact dynamically with the more formal social structures of the hospital. Ethnographic methods might be employed to understand the behaviours, dynamics, and communication of these groups in their natural settings, enabling a detailed appreciation of their functioning, content, and nature, adding important data to support these findings [40]. Further qualitative studies might also explore their outcomes and how participants interface with and move between the formal and informal domains, and potential unwanted or unforeseen consequences of these interactions. The alignment — or misalignment — of informal communities and formal care systems may carry important implications for clinical practice and policy, and this further understanding would inform health practitioners and health managers to optimally engage with these groups.

### Strengths and limitations

This qualitative study engaged with a wide range of participants with differing experiences, roles, and stages of treatment. Lines of questioning were focused on recent experiences of informal interactions, with the interviewer then asking the participants the meanings they attributed to these encounters, and how they may have understood other relationships and processes through treatment. The phenomenological approach, incorporated into both data collection and the analytical design, enabled methodological consistency, and a rich and textured account of the individual experience of engaging in informal interactions. The interviews were conducted by one researcher, yet analysis involved all the researchers, and began with foregrounding the pre-existing perceptions and interpretations of the interviewer, examining how these may have influenced initial interpretations, and the resultant coding and thematic structure. The structures of formal health care and social influences reflect the setting of the participants (in a developed country), influenced by local socio-cultural and health system factors, which may differ in other settings.

## Conclusions

Outside the confines of formal clinical discourses, patients, family carers, and staff members within the broad context of the hospital engage with each other in fluid, sometimes ephemeral, haphazard, and unpredictable ways. Within these informal interactions, individuals negotiate the terms of engagement, and share information, expertise, and personal stories, that they may employ to contribute to the individuals around them. The participants described relationships that could be profound, yet brief, or enduring and of limited depth, as well as others that have evolved to become integral to their social networks and support environments. Words such as support group, friendship, and community were often used to describe these engagements, and a wide range of meanings was elaborated that extended far beyond immediate cancer treatment. We propose that these interactions occur within a loose and evolving social network, an ‘informal community’, in which cancer patients, carers, and staff members play active and meaningful roles.

## Supplementary Information

Below is the link to the electronic supplementary material.

Supplementary file1 (DOCX 24 KB)

## Data Availability

This work has utilised interview data from staff, patients, and carers, which include the names of individuals that may be potentially identifiable. The data are thus not publicly available. The data may be made available upon reasonable request, dependant on their use, with enquiries directed towards the corresponding author (M. Grant).

## References

[1] Smith R. Smith R, editor. BMJ Opinion: BMJ. 2019. [cited 2020]. https://blogs.bmj.com/bmj/2019/02/07/richard-smith-the-public-health-of-death-dying-and-grief-has-been-neglected-but-now-is-the-time/. Accessed 13/07/2020

[2] Grant MP, Philip JA, Deliens L, Komesaroff PA (2019). Communal responsibility: a history of health collectives in Australia. Intern Med J.

[3] Isaksen AS, Gjengedal E (2000). The significance of fellow patients for the patient with cancer: what can nurses do?. Cancer Nurs.

[4] Timmermans S, Oh H (2010) The continued social transformation of the medical profession. J Health Soc Behav 51(1_suppl):S94-S106 10.1177/0022146510383500 20943586

[5] Foucault M (2012). The birth of the clinic.

[6] Larsen LS, Larsen BH, Birkelund R (2014). A companionship between strangers—the hospital environment as a challenge in patient–patient interaction in oncology wards. J Adv Nurs.

[7] Egestad H (2013). The significance of fellow patients for head and neck cancer patients in the radiation treatment period. Eur J Oncol Nurs.

[8] Wilson K, Luker KA (2006). At home in hospital? Interaction and stigma in people affected by cancer. Soc Sci Med.

[9] Birkelund R, Larsen LS (2013). Patient–patient interaction—caring and sharing. Scand J Caring Sci.

[10] Isaksen AS, Thuen F, Hanestad B (2003). Patients with cancer and their close relatives: experiences with treatment, care, and support. Cancer Nurs.

[11] Ludvigsen MS (2009). Patient life in hospital: a qualitative study of informal relationships between hospitalised patients.

[12] Goffman E (1961) Encounters: two studies in the sociology of interaction. Oxford, England: Bobbs-Merrill; 152

[13] Komesaroff P (2008). Experiments in love and death: medicine, postmodernism, microethics and the body.

[14] Drass KA (1982). Negotiation and the structure of discourse in medical consultation. Sociol Health Illn.

[15] Dieppe P, Rafferty A-M, Kitson A (2002). The clinical encounter—the focal point of patient-centred care. Health Expect: Int J Public Particip Health Care Health Policy.

[16] Laverty SM (2003). Hermeneutic phenomenology and phenomenology: a comparison of historical and methodological considerations. Int J Qual Methods.

[17] Tong A, Sainsbury P, Craig J (2007). Consolidated criteria for reporting qualitative research (COREQ): a 32-item checklist for interviews and focus groups. Int J Qual Health Care.

[18] Andersen LS, Larsen BH, Birkelund R (2015). A companionship between strangers—learning from fellow people with cancer in oncology wards. J Adv Nurs.

[19] Birkelund R, Larsen LS (2013). Patient-patient interaction—caring and sharing. Scand J Caring Sci.

[20] Bowen GA (2008). Naturalistic inquiry and the saturation concept: a research note. Qual Res.

[21] van Manen M (2017). But is it phenomenology?. Qual Health Res.

[22] Goffman E (1961) Encounters: two studies in the sociology of interaction: Ravenio Books;

[23] Girvan C, Conneely C, Tangney B (2016). Extending experiential learning in teacher professional development. Teach Teach Educ.

[24] Ng K-Y, Van Dyne L, Ang S (2009). From experience to experiential learning: cultural intelligence as a learning capability for global leader development. Acad Manag Learn Educ.

[25] Borkman T (1999) Understanding self-help/mutual aid: experiential learning in the commons: Rutgers University Press

[26] Rappaport J (1993). Narrative studies, personal stories, and identity transformation in the mutual help context. J Appl Behav Sci.

[27] Robinson D (1977) Self-help and health: mutual aid for modern problems. Martin Robertson, London

[28] Album D (1989) Patients’ knowledge and patients’ work. Patient-patient interaction in general hospitals. Acta Sociologica 32(3):295–306

[29] Larsen LS (2013). A companionship between strangers: patient-patient interaction in oncology wards.

[30] Larsen LS, Larsen BH, Birkelund R (2013). An ambiguous relationship—a qualitative meta-synthesis of hospitalized somatic patients’ experience of interaction with fellow patients. Scand J Caring Sci.

[31] Ridd M, Shaw A, Lewis G, Salisbury C (2009). The patient–doctor relationship: a synthesis of the qualitative literature on patients’ perspectives. Br J Gen Pract.

[32] Parsons T (1991). The social system.

[33] McDonnell O, Lohan M, Hyde A, Porter S (2009) Social theory, health and healthcare: Macmillan International Higher Education

[34] Bauman Z (2013) Liquid love: on the frailty of human bonds: John Wiley & Sons

[35] Bauman Z (1996) From pilgrim to tourist—or a short history of identity. In: Stuart Hall PdG, editor. Questions of cultural identity. 1: SAGE publications 18–36

[36] Walsh JC, High S (1999) Rethinking the concept of community. Histoire sociale/Social History

[37] Snowden D (2000) The social ecology of knowledge management. In: Despres C, Chauvel D, editors. The Present and the Promise of Knowledge Management. Woburn: Reed Elsevier

[38] Schulte B, Andresen F, Koller H (2020). Exploring the embeddedness of an informal community of practice within a formal organizational context: a case study in the German military. J Leadersh Org Stud.

[39] Wenger E (1998). Communities of practice: learning as a social system. Systems Thinker.

[40] Grant MP, Philip JA, Deliens L, Komesaroff PA (2022) Understanding complexity in care: opportunities for ethnographic research in palliative care. J Palliat Care 08258597221078375 10.1177/08258597221078375 35167402

