# Supplementary material for: ‘It’s communication between people who are going through the same thing’: experiences of informal interactions in hospital cancer treatment settings
Source: Support Care Cancer. 2023 Jul 3;31(7):440. doi: 10.1007/s00520-023-07900-6 (PMC10317905; doi:10.1007/s00520-023-07900-6)
Supplement: Supplementary file 1 — Supplementary file1 (DOCX 24 KB) [file 520_2023_7900_MOESM1_ESM.docx]

**Supplementary Files**

| Interview questions |
| --- |
| Patient and caregiver |
| 1. Can you tell me about the most recent conversation you had with someone here? 2. Can you tell me of another interaction you had with someone in the hospital last time you were here? 3. Tell me about the people you talk to when you come to hospital / day oncology? 4. Where do you get information about your cancer? 5. Have you accessed any support or internet groups for cancer? 6. Have you been connected with other people through family or friends who are undergoing treatment for cancer? 7. How would you describe yourself since your diagnosis? 8. How do you feel about the hospital environment as a place for talking to people? Could anything be altered? |
| Support group members |
| 1. Can you tell me about the people you met during treatment? 2. Can you tell me about a specific interaction that wasn’t planned? 3. How did you become involved with the support group? 4. Why do people come to these groups? 5. How was your group started? 6. Where do you get information about cancer? 7. Are you a member of other groups? 8. Have you been connected with other people through family or friends who are undergoing treatment for cancer? 9. How would you describe yourself since your diagnosis? 10. How might hospital be able to better support people going through cancer treatment? |
| Staff members |
| 1. Can you tell me about the most recent informal conversation you had with someone here? 2. Can you tell me of another interaction you had with someone in the hospital last time you were here? 3. Tell me about the interactions you have with patients and visitors 4. Tell me about some of the other interactions you see occurring 5. Do you feel this unit has a social role? 6. How is the set-up/environment of the unit? May it influence interactions? 7. What are your thoughts on how a diagnosis of cancer might influence an individual’s social functioning? 8. What are your perceptions of cancer support groups? Internet forums? |

**Table 1:** Question guides for participant groups

|  |  | n |
| --- | --- | --- |
| **Hospital patients** |  | 13 |
|  | Female | 8 |
|  | Age (mean) | 63.6 years |
| Primary cancer | Breast | 4 |
|  | Genito-urinary | 1 |
|  | Gastro-intestinal | 3 |
|  | Haematological | 4 |
|  | Gynaecological | 1 |
| Treatment intent | Curative | 2 |
|  | Chronic | 4 |
|  | Palliative | 7 |
|  |  |  |
| **Cancer support group members** |  | 5 |
|  | Female | 1 |
| Primary cancer | Genito-urinary | 4 |
|  | Breast | 1 |
|  |  |  |
| **Family carers** |  | 4 |
|  | Female | 2 |
|  |  |  |
| **Staff members** |  | 9 |
|  | Female | 7 |
| Formal role | Administrative | 2 |
|  | Nursing | 2 |
|  | Allied health | 2 |
|  | Doctor | 3 |

**Table 2** – Participant characteristics
